# Response: Clarifying the evidence and our position on universal school‐based mental health interventions: A response to Bonell et al.

**DOI:** 10.1111/camh.70125

**Published:** 2026-09-09

**Authors:** Jack L. Andrews, Lucy Foulkes

**Affiliations:** ^1^ Department of Experimental Psychology University of Oxford Oxford UK

**Keywords:** Prevention, intervention, universal, school, mental health

## Abstract

We appreciate the opportunity to respond to Bonell and colleagues' discussion of our work. However, the article does not accurately represent our position, attributing to us claims and arguments we have not made. There are five inaccuracies: the article (1) conflates arguments we have made in different papers; (2) generalises our arguments to all universal interventions when we specify therapeutically informed universal interventions; (3) claims we are definitive that universal interventions cause harm when we have stressed uncertainty around that word and concept; (4) states that we report meta‐analytic effect sizes incorrectly, but this is not the case; and (5) states that universal interventions are acceptable to young people, but evidence shows wide‐ranging, mixed responses. To offer clarity for readers, and in the interest of maintaining an accurate scientific record, we address these inaccuracies and reiterate what we originally wrote. We finish with a brief section summarising our position and the latest research. We hope that this clarification allows discussion to return to the pressing task of identifying which approaches meaningfully improve the mental health of young people, and which do not.

## Introduction

The literature on universal school‐based mental health interventions is extensive and complex. We therefore welcome detailed scrutiny and debate and recognise that different researchers, including Bonell and colleagues, may reasonably reach different conclusions about the evidence base and its implications for schools. However, productive debate depends on both positions being described correctly. The paper by Bonell and colleagues does not accurately represent our position (Bonell et al., 2026). The article at several points generates arguments we have not made by conflating distinct points made across two papers, generalises our position in multiple ways and incorrectly states that we misreported meta‐analytic estimates. These issues make it difficult for readers to distinguish genuine disagreements about the evidence, which are important and valid, from disagreements arising from an inaccurate representation of our position. Below, we list five inaccuracies in the paper by Bonell and colleagues, clarifying what we originally wrote, and end by summarising our current position.

1. The article conflates a broader hypothesis, from a different paper, with our work focusing on universal school mental health interventions

In 2023, we published a paper detailing the *prevalence inflation hypothesis*, in which we proposed that societal mental health awareness efforts might lead to an increase in reported mental health problems (Foulkes & Andrews, 2023). We proposed that this might happen via two routes: by leading people to report previously under‐reported symptoms (*improved recognition*) and by leading people to report milder forms of distress as symptomatic of a mental health problem (*overinterpretation*). We presented this as a hypothesis requiring empirical testing. We included school mental health interventions as one possible form of mental health awareness, among many others (public health campaigns, social media campaigns, etc.). The primary purpose of that paper was to propose a distinct mechanistic hypothesis about the impact of a changing public mental health conversation. It did not discuss or evaluate the overall utility of universal school mental health interventions, which we cover in a separate debate paper in 2025 drawing on distinct arguments (Andrews & Foulkes, 2025). However, Bonell and colleagues conflate these two bodies of our work, giving the wrong impression that our evaluation of universal school‐based interventions rests on the prevalence inflation hypothesis, which it does not. Our 2025 debate article critiqued by Bonell et al. neither cites the prevalence inflation paper nor relies on that hypothesis to reach its conclusions about universal school‐based interventions.

In addition, Bonell and colleagues state that we ‘offer three pieces of evidence to support the prevalence inflation hypothesis’, all of which are focused on school interventions, but this is inaccurate. In the original 2023 paper, the hypothesis was motivated by a broad body of indirect evidence spanning psychological debriefing, experimental studies, interventions and qualitative research, all of which are described in the original paper (Foulkes & Andrews, 2023). None of this evidence was presented as confirming prevalence inflation. Instead, it was used collectively to propose a hypothesis that, as we argued, requires testing.

Bonell and colleagues then transfer evidence and citations between our 2023 prevalence inflation paper and our 2025 school intervention debate paper, thereby generating arguments we have not made. First, they state that we cite a review of targeted and universal school anti‐bullying interventions (Guzman‐Holst, Zaneva, Chessell, Creswell, & Bowes, 2022) in our 2025 debate paper as evidence against universal school mental health interventions. We do not mention or cite this paper in our 2025 debate article. Rather, this review of anti‐bullying interventions was cited in our 2023 prevalence inflation hypothesis paper as one component of the broader body of indirect evidence motivating the need to test the hypothesis.

Second, Bonell and colleagues state that we use our scoping review of universal school‐based interventions based on CBT or mindfulness (Guzman‐Holst, Streckfuss Davis, Andrews, & Foulkes, 2025) as evidence of prevalence inflation. This is not correct. We cited this scoping review in our 2025 debate article as evidence that negative outcomes have been reported in some trials of school‐based mental health interventions. We did not cite it as evidence of prevalence inflation nor did we claim that the negative outcomes it identified arose through increased awareness. Indeed, within the 2025 Guzman‐Holst scoping review itself, we are explicit in making this point about these school interventions, stating that ‘It remains unclear whether these [negative] effects represent a genuine deterioration in an outcome that was supposed to improve, or if the intervention leads young people to report outcomes differently (for example due to increased awareness)’.

2 The article inaccurately states that our conclusions regarding negative effects apply to all universal school interventions

At several points, including in their abstract and introduction, Bonell and colleagues overgeneralise our position, again attributing to us arguments we have not made. In these instances, Bonell and colleagues characterise our position as opposing all universal school‐based interventions, a position we have not taken. In the abstract and main text of our 2025 debate paper, we specifically suggested a move away from ‘therapeutically informed universal school‐based mental health interventions’ (Andrews & Foulkes, 2025). In our introduction, we state that ‘we focus on therapeutically informed universal interventions, in which young people learn psychoeducational concepts and practical exercises that are taken from therapeutic modalities such as mindfulness, cognitive behavioural therapy (CBT) or dialectical behavioural therapy (DBT).’ This distinction is important and one which we have made consciously. The majority of evidence of negative effects comes from these specific types of universal interventions. We do not generalise to other intervention approaches, such as social and emotional learning, and we do not, as Bonell and colleagues write, ‘conclude that [all] universal interventions are insufficiently effective’ (Bonell et al., 2026).

3 The article inaccurately states that we are definitive about harm

Bonell and colleagues summarise us in multiple places, including in their abstract, as arguing that ‘many universal interventions to prevent mental‐health problems are harmful.’ This is another instance in which our position has been misrepresented and exaggerated. In our 2025 debate article, we have been careful and deliberate in our wording to state that therapeutically informed universal school‐based interventions have ‘the potential to elicit negative effects’ (Andrews & Foulkes, 2025). This distinction is important because not all negative effects constitute meaningful harm, just as not all positive effects constitute meaningful benefit. In other papers, we have used the term ‘potential harm’ to emphasise this uncertainty, arguing for the need to determine for whom negative effects might equate to harm, and the mechanisms through which this may occur (e.g. Foulkes, Holst, & Andrews, 2026). Identifying harm as a possibility requiring investigation is not the same as concluding that harm has been demonstrated.

4 The article inaccurately states that we misreport effect sizes from a key meta‐analysis, and then draws population‐level conclusions from incorrect effect sizes

One specific point raised by Bonell and colleagues rests on a claim that we have incorrectly reported long‐term (>12 months) effect sizes of universal school‐based interventions from Werner‐Seidler et al. (2021). We stand by our reported figures, which show that the long‐term effect sizes for universal school‐based interventions in that meta‐analysis are below .10 for both depression (*g* = 0.07) and anxiety (*g* = 0.09). We refer readers to page 12, section 4.2.1 of the Werner‐Seidler and colleagues' paper. The figures quoted by Bonell and colleagues (*g* = 0.10 for depression and *g* = 0.11 for anxiety) instead refer to long‐term estimates combined across universal *and targeted* interventions, not universal interventions alone, and are therefore the wrong numbers to report when discussing universal interventions. The Werner‐Seidler meta‐analysis reviews targeted and universal interventions with a range of content; it is not a review of universal CBT interventions as stated by Bonell and colleagues.

Bonell and colleagues then use these incorrectly attributed effect sizes to make an argument in support of Rose's prevention framework, an important public health principle which hypothesises that small reductions in risk, when distributed across a population, may produce meaningful population benefit. However, this principle is only relevant for interventions that have a sufficiently reliable positive effect to scale up. The correct long‐term effect sizes for universal interventions reported by Werner‐Seidler and colleagues are smaller than those quoted by Bonell and colleagues, and the 95% confidence intervals around both the depression and anxiety estimates cross zero, leaving open the possibility that the true average long‐term effect is null. Even if we were confident of a small positive average effect, evidence indicates that intervention effects from universal school interventions may vary according to baseline risk. Indeed, Bonell and colleagues themselves write in their first section that ‘much of the evidence of harm is from subgroups with *worse* prior mental health.’ Collectively, these points raise uncertainty and important ethical concerns when considering the applicability of Rose's hypothesis to universal school mental health interventions.

5 The article inaccurately states that universal interventions are acceptable to young people

We agree with Bonell and colleagues' critique that conclusions about acceptability should be drawn from a larger body of qualitative work than the two studies we cite, and further agree that multiple qualitative studies show acceptability among some young people and not among others. However, we do not agree with Bonell and colleagues' conclusion that ‘broader qualitative evidence suggests that universal school interventions are acceptable’. Bonnell and colleagues cite a 2022 NICE evidence synthesis to support this, but the authors of that review concluded that there was ‘a limited evidence base of qualitative data’ and that the evidence ‘should not be the sole basis for any recommendations.’ In addition, perceptions of programme impact among children from the NICE review drew on five studies, four of which focused on interventions delivered in primary school settings (NICE, 2022). Acceptability may vary across development, and findings from primary school children may not be generalisable to secondary school adolescents. This is important because our argument in our 2025 debate article centred on adolescents, with our evidence coming from secondary school contexts.

Studies published since 2022 evaluating therapeutically informed universal interventions among adolescents have demonstrated a more mixed picture. Several recent studies have shown the same findings as the two papers we cited: that some adolescents find these interventions relevant, helpful and informative and some find them boring, distressing and confusing (full list of papers available on request). A more accurate conclusion of the available evidence, including the most recent studies, is that young people vary widely in their experiences of universal mental health interventions, particularly those that are therapeutically informed.

## Conclusion

The evidence on universal school‐based mental health interventions is extensive and heterogenous, which has understandably left room for multiple interpretations. In this final section, we clarify our own interpretation of the evidence base.

Our first conclusion is that current meta‐analytic evidence does not reliably or consistently demonstrate that universal school‐based mental health interventions improve young people's mental health. For example, the recent Hayes et al. (2025) review of universal interventions found small positive average effects post intervention for anxiety and depression, but none for more general internalising symptoms. However, the review found substantial heterogeneity in effect sizes and possible publication bias among depression and internalising outcomes, with the authors citing caution when interpreting the findings. Crucially, only 9 of the 71 studies were judged by the authors to be of high quality. This introduces uncertainty. Given that some recent trials have also shown null and negative effects in the short and longer term, the available evidence needs to be contextualised within these limitations.

Second, it is also undeniable now that negative effects are a possible outcome of universal interventions. These have occurred most often in therapeutically informed interventions (Yao et al., 2026), although recent broader mental health awareness interventions also reported negative effects. The open question is whether negative effects are sufficiently frequent or concerning enough to influence decisions about school mental health provision, leaving room for differences of opinion. In our scoping review of group‐based CBT and mindfulness mental health interventions, we found that 8.93% of these interventions reported at least one negative effect. However, when the evidence was restricted to high quality studies this number rose to 33.3%, all in universal interventions (Guzman‐Holst et al., 2025). Similarly, a more recent review of universal school‐based mental health interventions found potential harm in 29% of all included studies, with significant deterioration reported more often among CBT‐ and mindfulness‐based trials (Yao et al., 2026). These findings need to be interpreted alongside an evidence base demonstrating that these interventions do not reliably improve mental health outcomes and qualitative work indicating that, for at least some young people, these lessons really are experienced negatively. Given this, we believe it is legitimate to investigate this and reasonable to recommend caution around delivering these interventions universally in schools, particularly those that are therapeutically informed.

Given that universal school‐based interventions will potentially reach every young person, their implementation warrants an evidential standard proportionate to that reach. In our view, the current evidence for therapeutically informed universal school‐based mental health interventions does not meet this standard. Instead of these programmes, time and effort are likely better spent focusing on evidence‐based, targeted or indicated interventions, delivering more personalised support to young people who want or need it.

## Author contributions

J.L.A. and L.F. contributed equally.

## Conflict of interest statement

The authors declare no conflicts of interest.

## Funding information

J.L.A. is funded by Wellcome (WT/227640/Z/23/Z) and L.F. is funded by the Prudence Trust.

## AI statement

The authors declare that they have not used AI in any capacity that would require disclosure in accordance with Wiley's AI Guidelines nor in any capacity that would reasonably require disclosure for editors, reviewers and readers to properly evaluate their research or manuscript. The authors confirm that AI has not been used for purposes including but not limited to drafting and editing, to generate substantial text or restructure arguments, nor in the research methodology. The authors take full responsibility for the accuracy of this statement.

## Ethical considerations

Ethics approval is not applicable to this commentary article.

## Data Availability

Data sharing is not applicable to this article as no datasets were generated or analysed for this commentary article.
